# MYCN amplification plus 1p36 loss of heterozygosity predicts ultra high risk in bone marrow metastatic neuroblastoma

**DOI:** 10.1002/cam4.4583

**Published:** 2022-02-09

**Authors:** Zhi‐Xia Yue, Tian‐Yu Xing, Wen Zhao, Qian Zhao, Xi‐Si Wang, Yan Su, Chao Gao, Shu‐Guang Liu, Xiao‐Li Ma

**Affiliations:** ^1^ Hematologic Disease Laboratory, Hematology Center, Beijing Key Laboratory of Pediatric Hematology Oncology; National Key Discipline of Pediatrics (Capital Medical University); Key Laboratory of Major Diseases in Children, Ministry of Education Beijing Pediatric Research Institute, Beijing Children's Hospital, Capital Medical University, National Center for Children's Health Beijing China; ^2^ Medical Oncology Department, Pediatric Oncology Center, Beijing Children's Hospital Capital Medical University, National Center for Children's Health Beijing China; ^3^ Beijing Key Laboratory of Pediatric Hematology Ocology, Key Laboratory of Major Diseases in Children Ministry of Education Beijing China

**Keywords:** 11q23 LOH, 1p36 LOH, bone marrow metastasis, fluorescence in situ hybridization, MYCN amplification, neuroblastoma, survival

## Abstract

**Background:**

This study aimed to better understand the prognostic effect of multiple genetic markers and identify more subpopulations at ultra high risk of poor outcome in bone marrow (BM) metastatic neuroblastoma (NB).

**Methods:**

We screened the MYCN, 1p36 and 11q23 loss of heterozygosity (LOH) statuses of 154 patients by interphase fluorescence in situ hybridization of BM cells. The clinical characteristics of patients with the three markers and their associations with prognosis were analysed.

**Results:**

MYCN amplification and LOH at 1p36 and 11q23 were identified in 16.2%, 33.1% and 30.5% of patients, respectively. There were strong associations between MYCN amplification and 1p36 LOH as well as 11q23 LOH. Both MYCN amplification and 1p36 LOH were strongly associated with high levels of lactate dehydrogenase (LDH) and neuron‐specific enolase, more than 3 metastatic organs, and more events. 11q23 LOH occurred mainly in patients older than 18 months, and those who had high LDH levels. In univariate analysis, patients with MYCN amplification had poorer prognosis than those without. Patients with 1p36 LOH had a 3‐year event‐free survival (EFS) and overall survival lower than those without. 11q23 LOH was associated with poorer EFS only for patients without MYCN amplification. In a multivariate model, MYCN amplification was independently associated with decreased EFS in all cohorts. 11q23 LOH was an independent prognostic factor for patients without MYCN amplification, whereas 1p36 LOH was not an independent marker regardless of MYCN amplification. Compared with all cohorts, patients with both MYCN amplification and 1p36 LOH had the worst outcome and clinical features.

**Conclusions:**

Patients with both MYCN amplification and 1p36LOH had the worst survival rate, indicating an ultra high‐risk group. Our results may be applied in clinical practice for accurate risk stratification in future studies.

## Graphical abstract

Our work identified patients with both MYCN amplification and 1p36 loss of heterozygosity had the worst outcome indicating ultra high‐risk. Moreover, it also demonstrated their associations and clinicobiological characteristics. The results can be applied to clinic and ultimately improve the long‐time survival.

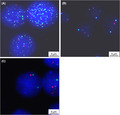

## 1 BACKGROUND

Neuroblastoma (NB) represents the most frequent extracranial solid tumour in early childhood.^1^ The early clinical manifestations are extremely insidious. More than 50% of patients are diagnosed after metastasis, and bone marrow (BM) is the preferential site in which it presents in more than 80% of metastatic NBs.^2^, ^3^ In addition, BM is also a frequent site of disease recurrence,^4^ thus making it particularly attractive as a means with which to evaluate disease response.

According to the International Neuroblastoma Risk Group (INRG) classification system, patients were divided into very low‐, low‐, intermediate‐ and high‐risk groups.^5^ More than 80% of patients with BM metastatic NB are at high‐risk and have a poor prognosis.^6^ However, due to the broad heterogeneity of tumours, many groups have attempted to further define a subgroup of high‐risk patients with a particularly increased risk of poor outcome. To date, there is no standard consensus in the definition of these ultra high‐risk patients.^7^ The biological characteristics and more front‐line therapeutic alternatives still need to be explored.

Tumour genetics symbolise an important area for risk stratification of high‐risk NB.^8^ For example, MYCN amplification is the most powerful marker for a refractory phenotype and high risk of relapse.^9^ Whereas MYCN amplification is only present in approximately 20% of all NB cases, more than 60% of high‐risk NB cases do not have this aberration.^10^ Loss of heterozygosity (LOH) at 1p36 and 11q23 are frequent segmental chromosomal losses found in NB.^8^ The mechanism of their influence on oncogenesis and tumour progression may be related to the loss of tumour suppressors at corresponding chromosomal regions.^11^ Although the prognostic effect of a single genetic abnormality has been studied in some trials, the combined effect and their roles in identifying ultra high‐risk NB are still not clear.

To better understand the prognostic effect of multiple genetic markers and identify more subpopulations at ultra high risk of poor outcome, this study determined the status of the MYCN gene, 1p36 and 11q23 simultaneously in a large series of BM metastatic NB, compared their clinical features and analysed the survival situations separately and together. Our aim was to further stratify high‐risk patients, and the results provide an important basis for adjusting treatment regimens in the future.

## 2 MATERIALS AND METHODS

### 2.1 Patients

A total of 154 consecutive paediatric patients with newly diagnosed NB at the Hematology Oncology Center in Beijing Children's Hospital affiliated with Capital Medical University from January 2016 to December 2018 were enrolled for this research.

All patients had BM metastasis with more than 20% NB cells in the diagnostic BM samples. The patients were diagnosed according to the International Neuroblastoma Risk Group Staging System (INRGSS)^12^ and treated with the BCH‐NB‐2007 protocol (based on the Hong Kong Paediatric Haematology and Oncology Study Group guidelines and the results of a study in Germany)^.^ ^13^ All patients had received multidisciplinary treatment according to risk stratification. After that, the patients were monitored and evaluated every 3 months. Therapeutic responses were mainly determined by microscopic examination of BM tissues, levels of serum tumour markers, computed tomography or magnetic resonance imaging, and ultrasound. The final date for data collection was March 2020, with a median follow‐up time of 24 months (range 0.3–51 months). This research was approved by the Beijing Children's Hospital Institutional Ethics Committee (No. 2016‐65). Informed consent was obtained from the patients' parents or their guardians.

### 2.2 Treatment

The BCH‐NB‐2007 protocol for low‐/intermediate‐risk NB including 2–3 cycles of the CBVP regimen (carboplatin [200 mg/m^2^] and etoposide [150 mg/m^2^]) and the CADO regimen (vincristine [1.5 mg/m^2^] and adriamycin [25 mg/m^2^]) alternately used. Then, the primary tumour was resected. If the patient had surgery first, 2–3/4–6 cycles of chemotherapy for low‐/intermediate‐risk patients were needed after surgery. The treatment protocol for high‐risk NB included seven cycles of intensive chemotherapy: CAV regimen [cyclophosphamide (70 mg/kg) adriamycin (25 mg/m^2^) and vincristine (0.033 mg/kg)] for cycles 1, 2, 4 and 6 and CVP regimen [cisplatin (50 mg/m^2^) and VP16 (200 mg/m^2^) for cycles 3, 5 and 7. After four cycles of chemotherapy, the primary tumour was resected. Patients received autologous peripheral blood stem cell (PBSC) transplantation after seven cycles of chemotherapy. Two cycles of chemotherapy were added if patients could not receive transplantation. The primary site received local irradiation (20–25 Gy). The maintenance chemotherapy protocol was 13‐*cis*‐retinoic acid (160 mg/m^2^/day) for 9–12 months.

Patients in the high‐risk group needed to receive transplantation, except for the following cases: disease that progressed before the time of transplantation, too many postoperative complications to receive transplantation, receipt of immunotherapy, tumour too large to be completely removed, and economic difficulties. In our study, 71 cases received transplantation among the 146 high‐risk patients.

### 2.3 Sample processing and biologic studies

BM samples were stored in heparin‐coated tubes by BM puncture at diagnosis. The cells were cultured in RPMI‐1640 (HyClone) and foetal bovine serum medium (Invitrogen) for 24 h at a ratio of 4:1. The final cell density was (1–3) × 10^6^/ml. After culture, cells were treated with 0.075 mol/L KCl for 30 min and fixed twice in a 3:1 mixture of methanol: acetic acid for 20 min. The cell suspension was preserved in a fresh fixative solution at 4°C for further fluorescence in situ hybridization (FISH) examination. Morphological examination of BM biopsy was performed by at least two independent pathologists to determine the presence of NB cells. Venous blood samples were collected at the time of diagnosis for the detection of specific tumour markers, including lactate dehydrogenase (LDH) and neuron‐specific enolase (NSE), by radioimmunoassay and full‐automatic biochemical analysis.

### 2.4 FISH analysis of BM cells

Interphase FISH in a dual‐colour procedure was performed as previously reported ^[15]^. The status of MYCN was determined using a DNA probe from Vysis [N‐MYC(2p24)/CEP2(2p11.1‐q11.1) Dual Colour Probe] (cat. No. 7J72‐01, Abbott Laboratories). LOH at 1p36 and 11q23 was determined with DNA probes from Anbiping [GSP1q23/SRD(1p36)] and [CSP11/KMT2A(11q23)] (Cat.F.01025 and 01308, Anbiping). DAPI was used to counterstain the nuclei, and 500 cells were counted on each slide. Fluorescence images were collected with a Leica DM6000B microscope (Leica Microsystems GmbH). Interpretations for the above alterations were performed according to the guidelines of the European Neuroblastoma Quality Assessment group.^15^ Briefly, MYCN amplification was defined as a fourfold increase in MYCN signals in relation to the number of chromosome 2. Both 1p36 LOH and 11q23 LOH were defined as only one signal of the target gene compared with the two signals of the reference gene.

### 2.5 Statistical analysis

SPSS software, version 16.0 (SPSS Inc.) was used for statistical analyses in this study. Tests of association were evaluated using the χ^2^ test. Survival analyses were performed according to the Kaplan–Meier method, and comparisons of event‐free survival (EFS) and overall survival (OS) were constructed with a two‐sided log‐rank test. EFS was defined as the time from the date of diagnosis until the date of one of the following events: relapse, progression and death (whichever came first). Patients in continuous complete remission (CR) were censored at the date of last contact. OS was estimated from the diagnostic date to the date of death due to any reason, or the last contact with patients in continuous CR. Multivariate analyses were performed with the Cox proportional hazards regression model to identify the independent prognostic factors influencing EFS, and variables reaching a *p* < 0.05 in the respective univariate analysis were included in the model. For all tests, two‐sided *p* < 0.05 was considered statistically significant.

## 3 RESULTS

### 3.1 Clinical characteristics of patients with BM metastatic NB

The clinicobiological characteristics of the 154 patients with BM metastatic NB were detailed in Table 1. There were slightly more males than females, with a ratio of 1.3:1. The median age was 43 months (1–148 months), and 87.7% were older than 18 months. Most patients (74.1%) had normal white blood cell (WBC) counts, with a range of 1.74–42.52 × 10^9^/L (median, 6.035 × 10^9^/L). There were 136 patients (87%) with moderate to severe anaemia, with a median haemoglobin level of 96 g/L (range 58–132 g/L). Approximately half the patients had thrombocytopenia ranging from 37 × 10^9^/L to 669 × 10^9^/L (median, 269.5 × 10^9^/L). Most of the patients (94.8%) were in the high‐risk group, and the abdomen was the most common primary site of the tumour (87.0%). Among the patients, only 20.1% had very high LDH levels (≥1500 IU/L), and 46.8% had very high NSE levels (≥370 ng/ml). The tumour diameters ranged from 1.5 to 23 cm (median, 10.55 cm), and 85 patients (55.2%) had tumour diameters >10 cm at diagnosis. More than half of the patients were initially diagnosed with the largest tumour diameter of more than 10 cm. Metastasis of more than three organs occurred in 32 cases, accounting for 20.8% of the patients. In addition, bone was the most frequent metastatic site. After a median follow‐up time of 24 months (range 0.3–51 months), 42 (27.3%) patients died, 30 (19.5%) patients suffered from progression or tumour relapse, and the remaining 82 (53.2%) had stable disease. The three‐year EFS and OS for the entire cohort were 51.8 ± 4.3% and 68.4 ± 4.4%, respectively.

**TABLE 1:** Clinical features of patients with bone marrow metastatic NB

Characteristics	Total patients *n* (%)	Patients with both MYCN amplification and 1p36 LOH *n* (%)	*p*‐values^a^
Number	154	17	
Sex			
Male	87 (56.5)	11 (64.7)	0.611
Female	67 (43.5)	6 (35.3)	
Age (months)			
<18	19 (12.3)	1 (5.9)	0.744
18–60	98 (63.7)	11 (64.7)	
≥60	37 (24.0)	5 (29.4)	
Median	43	43	
Range	1–148	12–105	
WBC (×10^9^/L)			
<4	25 (16.2)	4 (23.5)	0.626
4–10	114 (74.1)	11 (64.7)	
≥10	15 (9.7)	2 (11.8)	
Haemoglobin (g/L)			
<60	2 (1.3)	1 (5.9)	0.472
60–90	49 (31.8)	5 (29.4)	
90–120	85 (55.2)	10 (58.8)	
≥120	18 (11.7)	1 (5.9)	
Platelet (×10^9^/L)			
<100	14 (9.1)	7 (41.2)	<0.001
100–300	82 (53.2)	9 (52.9)	
≥300	58 (37.7)	1 (5.9)	
Risk stratification			
Low‐risk	2 (1.3)	0	–
Intermediate‐risk	6 (3.9)	0	
High‐risk	146 (94.8)	17 (100%)	
Primary site			
Abdomen	134 (87.0)	16 (94.1)	1.0
Thorax	16 (10.4)	1 (5.9)	
Others	4 (2.6)	0	
LDH (IU/L)			
<1500	123 (79.9)	2 (11.8)	<0.001
≥1500	31 (20.1)	15 (88.2)	
NSE (ng/ml)			
<370	82 (53.2)	1 (5.9)	<0.001
≥370	72 (46.8)	16 (94.1)	
*MYCN* gene			
Amplified	25 (16.2)	17 (100)	–
Nonamplified	129 (88.1)	0	
11q23 LOH			
LOH	47 (30.5)	0	–
No loss	107 (69.5)	17 (100)	
1p36 LOH			
LOH	51 (33.1)	17 (100)	–
No loss	103 (66.9)	0	
Tumour size (cm)			
<10	69 (44.8)	6 (35.3)	0.608
≥10	85 (55.2)	11 (64.7)	
Number of organs with metastasis			
<3	86 (57.1)	3 (17.6)	0.005
3	35 (22.1)	6 (35.3)	
>3	33 (20.8)	8 (47.1)	
Metastatic site			
Bone	121 (78.6)	15 (88.2)	0.456
Distant lymph node	97 (63.0)	13 (76.5)	
Central nervous system	37 (24.0)	9 (52.9)	
Others	75 (48.7)	13 (76.5)	
Event			
No event	82 (53.2)	2 (11.8)	<0.001
Relapse/progression	30 (19.5)	0	
Death	42 (27.3)	15 (88.2)	
Survival time			
Median EFS time	19.0	12.0	<0.001
Median OS time	24.0	14.0	<0.001

Abbreviations: LDH, lactate dehydrogenase; LOH, loss of heterozygosity; NB, neuroblastoma; NSE, neuron‐specific enolase; WBC, white blood cell.

^a^ The χ^2^ test was performed for statistical analysis.

We also analysed the characteristics of 17 patients with both MYCN amplification and 1p36 LOH (Table 1). All 17 patients were in the high‐risk group, and no 11q23LOH was found in these patients. Compared with all patients, there were significant differences in the presence of adverse prognostic factors, including high LDH and NSE levels (*p* < 0.001, *p* < 0.001), metastatic site of three or more (*p* = 0.004), and poor treatment effect (*p* < 0.001). Thrombocytopenia was also more common in these patients than in the entire cohort (*p* < 0.001). Although there was no difference in the metastasis site, the proportion of central nervous system metastasis in patients with both MYCN amplification and 1p36 LOH was high. The median survival times of EFS and OS were significantly shorter than those of all cohorts (both *p* < 0.001).

### 3.2 Frequency and relationship of genetic markers in BM cells

**FIGURE 1:**
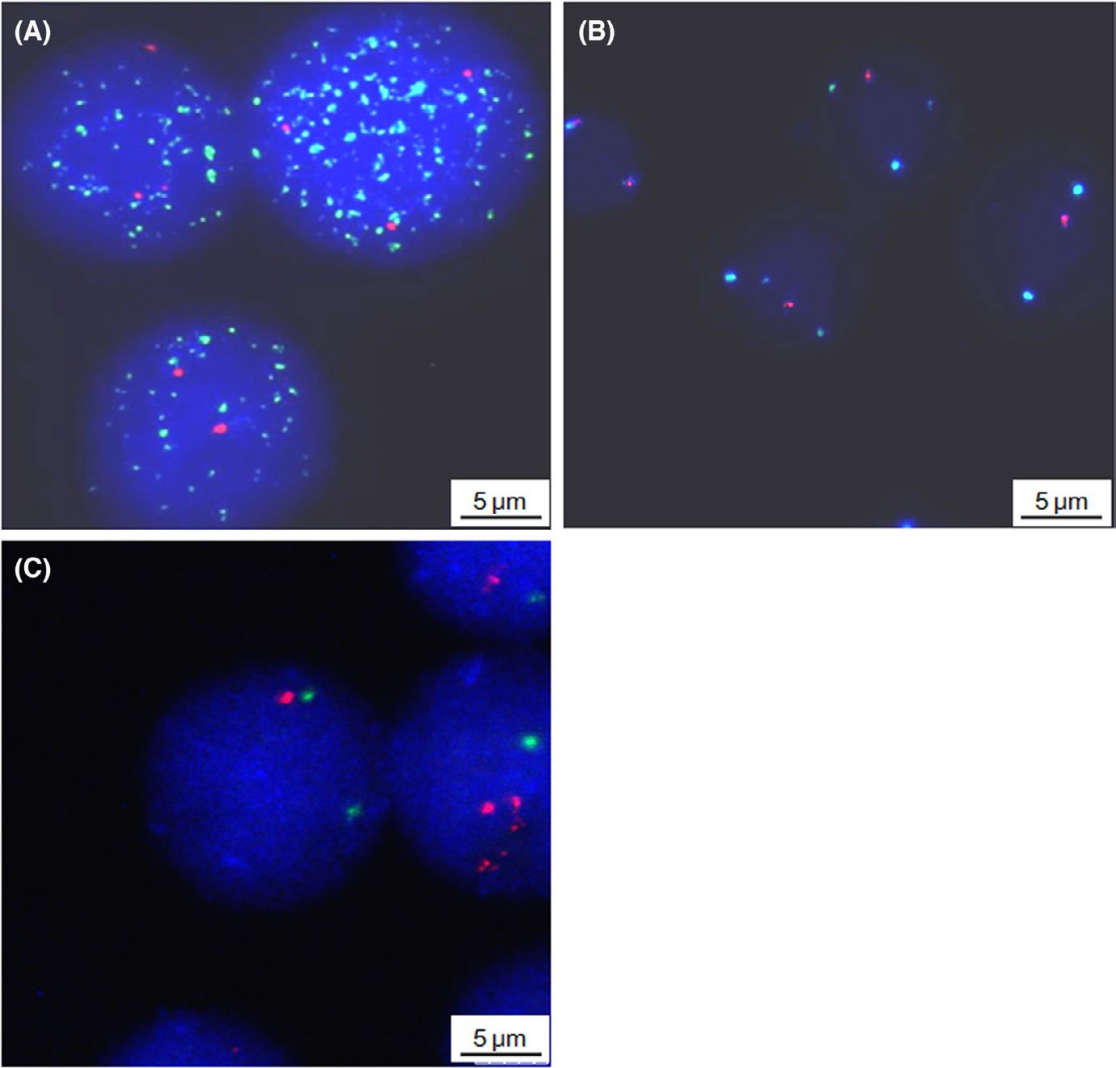
Representative interphase FISH images of bone marrow cells. (A) The image represents a case with MYCN amplification using a MYCN (2p24) (green) and chromosome 2 centromere (red) DNA probe. Three centromeric signals and more than 50 copies MYCN signals were seen within the nuclei. (B) The image of a case with 11q23 LOH using a CSP11 (green)/KMT2A (11q23) (red) DNA probe. The nuclei show two chromosome 11 signals and only one KMT2A (11q23) signal. (C) The image shows a case with 1p36 LOH using a GSP1q23 (green)/SRD (1p36) (red) DNA probe. The nucleus in the middle has two green signals and only one red signal. FISH, fluorescence in situ hybridization; LOH, loss of heterozygosity

In terms of genetic aberrations, MYCN amplification was seen in 25 cases (16.2%). LOH at 11q23 and 1p36 was detected in 47 (30.5%) and 51 (33.1%) cases respectively (Figure 1). There were significant associations between MYCN amplification and 1p36 LOH (*p* < 0.001) as well as MYCN amplification and 11q23 LOH (*p* = 0.001) (Table 2). However, there was no association between 11q23LOH and 1p36 LOH (*p* = 0.062).

**TABLE 2:** Frequency and association of MYCN amplification, 1p36 LOH and 11q23 LOH in bone marrow cells detected by FISH

Aberrations	1p36 status *n* (%)		*p* value	11q23 status *n* (%)		*p* value^a^
	LOH no loss			LOH no loss		
*MYCN* gene			<0.001			0.001
Amplified	17 (33.3)	8 (7.8)		1 (2.1)	24 (22.4)	
Nonamplified	34 (66.7)	95 (92.2)		46 (97.9)	83 (77.6)	
11q23 LOH			0.062	NA		
LOH	21 (41.2)	26 (25.2)				
No loss	30 (58.8)	77 (74.8)				

Abbreviation: LOH, loss of heterozygosity.

^a^The χ^2^ test was performed for statistical analysis.

**FIGURE 2:**
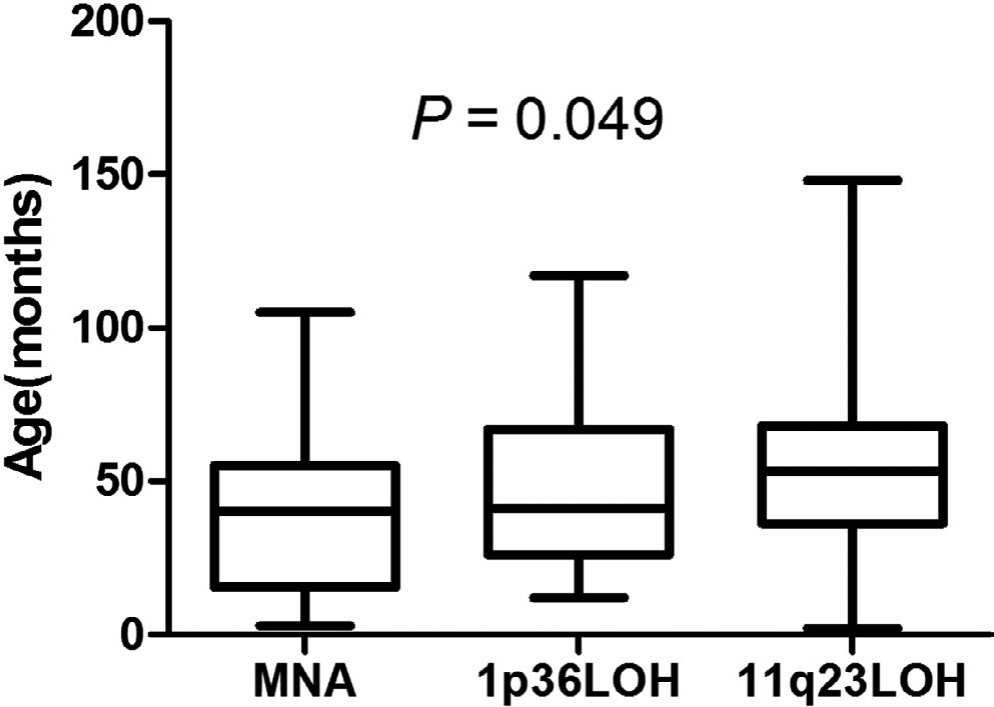
The initial age of diagnosis of the MYCN‐amplified (MNA),1p36 LOH and 11q23 LOH group. LOH, loss of heterozygosity

An obvious difference in age at diagnosis was seen among the MYCN‐amplified (MNA),1p36LOH and 11q23LOH groups (Figure 2). The median age at diagnosis was 40 months in the MYCN‐amplified group, 41 months in the 1p36 LOH group and 53.3 months in the 11q23 LOH group (*p* = 0.049).

### 3.3 Comparison of clinicobiological characteristics according to the status of MYCN, 1p36 and 11q23

There were obvious associations between MYCN amplification and high levels of LDH as well as NSE (*p* < 0.001, *p* < 0.001), more than three organs with metastasis (*p* = 0.004), and poor treatment effect with more events occurring (*p* < 0.001). 11q23 LOH was significantly associated with age of more than 18 months (*p* = 0.041), and high LDH levels (*p* = 0.028). 1p36 LOH was significantly associated with high LDH and NSE levels (*p* < 0.001, *p* = 0.002), more than three organs with metastasis (*p* = 0.038) and a poor treatment effect (*p* = 0.002) (Table 3).

**TABLE 3:** Comparison of clinicobiological characteristics according to the status of MYCN, 1p36 and 11q23 (*n* = 154)

Variables	*MYCN* status			11q23 status			1p36 status		
	Amplified *n* (%)	Nonamplified *n* (%)	*p*‐values^a^	LOH *n* (%)	No loss *n* (%)	*p*‐values^a^	LOH *n* (%)	No loss *n* (%)	*p*‐values^a^
Total	25	129		47	107		51	103	
Gender									
Male	17 (68.0)	70 (54.3)	0.271	32 (68.1)	55 (51.4)	0.077	30 (58.8)	57 (55.3)	0.732
Female	8 (32.0)	59 (45.7)		15 (31.9)	52 (48.6)		21 (41.2)	46 (44.7)	
Age (months)									
<18	7 (28.0)	12 (9.3)	0.05	2 (4.3)	17 (15.9)	0.041	3 (5.9)	16 (15.5)	0.221
18–60	13 (52.0)	85 (65.9)		29 (61.7)	69 (64.5)		34 (66.7)	64 (62.2)	
≥60	5 (20.0)	32 (24.8)		16 (34.0)	21 (19.6)		14 (27.4)	23 (22.3)	
Primary site									
Abdomen	23 (92.0)	111 (86.1)	0.378	38 (80.9)	96 (89.7)	0.276	44 (86.3)	90 (87.4)	0.270
Thorax	1 (4.0)	15 (11.6)		7 (14.9)	9 (8.4)		7 (13.7)	9 (8.7)	
Others	1 (4.0)	3 (2.3)		2 (4.2)	2 (1.9)		0 (0)	4 (3.9)	
LDH (IU/L)									
<1500	7 (28.0)	116 (89.9)	<0.001	43 (91.5)	80 (74.8)	0.028	31 (62.7)	92 (89.3)	<0.001
≥1500	18 (72)	13 (10.1)		4 (8.5)	27 (25.2)		20 (37.3)	11 (10.7)	
NSE (ng/ml)									
<370	5 (20.0)	77 (59.7)	<0.001	21 (44.7)	61 (57.0)	0.166	18 (35.3)	64 (62.1)	0.002
≥370	20 (80.0)	52 (40.3)		26 (55.3)	46 (43.0)		33 (64.7)	39 (37.9)	
Tumour size (cm)									
<10	9 (36.0)	60 (46.5)	0.385	19 (40.4)	50 (46.7)	0.863	19 (37.3)	50 (48.5)	0.229
≥10	16 (64.0)	69 (53.5)		28 (59.6)	57 (53.3)		32 (62.7)	53 (51.5)	
Number of organs with metastasis									
<3	7 (28.0)	79 (61.2)	0.004	25 (53.2)	61 (57.0)	0.726	22 (43.1)	64 (62.1)	0.038
≥3	18 (72.0)	50 (38.8)		22 (46.8)	46 (43.0)		29 (56.9)	39 (37.9)	
Event									
No event	4 (16.0)	78 (60.5)	<0.001	23 (48.9)	59 (55.1)	0.118	18 (35.3)	64 (62.1)	0.002
Event	21 (84.0)	51 (39.5)		24 (51.1)	48 (44.9)		33 (64.7)	39 (37.9)	

Abbreviations: LDH, lactate dehydrogenase; LOH, loss of heterozygosity; NSE, neuron‐specific enolase.

^a^ The χ^2^ test was performed for statistical analysis.

### 3.4 Effect of MYCN amplification and 1p36 and 11q23 LOH on the prognosis of BM metastatic NB

**FIGURE 3:**
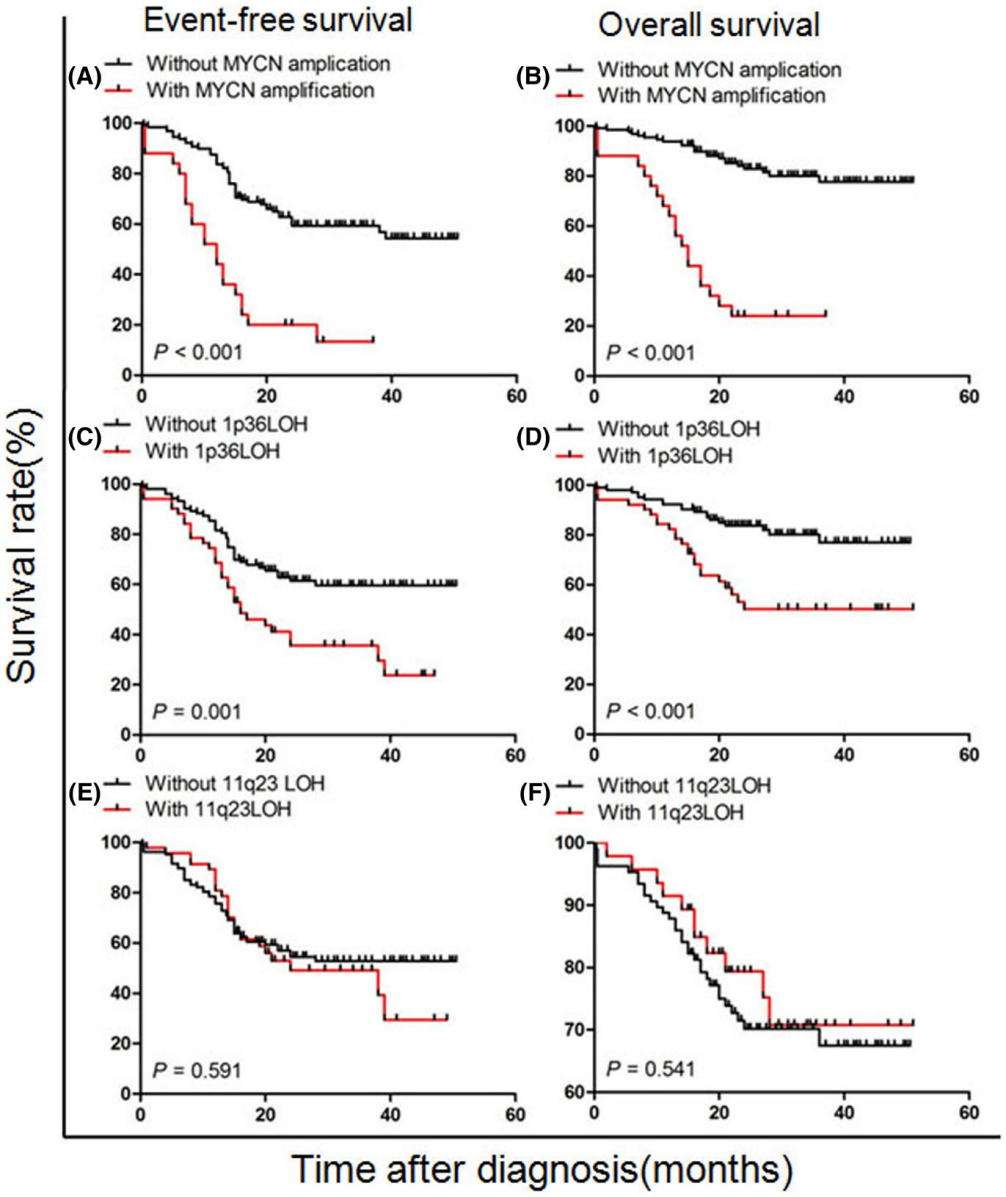
Survival rate according to MYCN amplification, 1p36 and 11q23 LOH in all patients with bone marrow metastatic neuroblastoma. (A, B) Event‐free survival (EFS) and overall survival (OS) are stratified by MYCN amplification. (C, D) EFS and OS stratified by 1p36 LOH. (E, F) EFS and OS are stratified by 11q23 LOH. LOH, loss of heterozygosity

Univariate analysis of patient outcomes showed that MYCN amplification and 1p36LOH were significantly associated with a decreased survival rate. Patients with MYCN amplification had 3‐year EFS and OS rates of 13.3 ± 7.6% and 24.0 ± 8.5%, respectively, compared with 54.4 ± 5.4% (*p* < 0.001) and 77.6 ± 4.5% (*p* < 0.001) in patients who did not have MYCN amplification (Figure 3A,B). Patients with 1p36LOH had 3‐year EFS and OS rates of 23.8 ± 8.4% and 50.2 ± 7.7%, respectively, compared with 59.7 ± 5.2% (*p* = 0.001) and 76.9 ± 5.2% (*p* < 0.001) in patients without 1p36LOH (Figure 3C,D). There was no significant difference in survival between patients with and without 11q23 LOH (Figure 3E,F) (Table 4).

**TABLE 4:** Univariate analysis of event‐free survival and overall survival

Cohort and marker	Total	3‐year EFS (%)	*p*	3‐year OS (%)	*p*
All patients					
MYCN status					
Amplified	25	13.3 ± 7.6	<0.001	24.0 ± 8.5	<0.001
Nonamplified	129	54.4 ± 5.4		77.6 ± 4.5	
1p36 status					
LOH	51	23.8 ± 8.4	0.001	50.2 ± 7.7	<0.001
No loss	103	59.7 ± 5.2		76.9 ± 5.2	
11q23 status					
LOH	47	29.5 ± 11.8	0.591	70.8 ± 8.0	0.541
No loss	107	52.9 ± 5.1		67.5 ± 5.2	
MYCN nonamplified					
11q23 status					
LOH	46	28.9 ± 1.6	0.025	70.3 ± 8.0	0.127
No loss	83	65.4 ± 5.5		81.8 ± 5.2	
1p36 status					
LOH	34	31.8 ± 11.1	0.095	71.3 ± 8.9	0.26
No loss	95	63.4 ± 5.2		79.5 ± 5.3	

*Note*: Univariate analysis for EFS and OS were calculated with the use of Kaplan–Meier log‐rank test. All factors with *p* < 0.05 in the univariate analysis are shown.

Abbreviations: EFS, event‐free survival; LOH, loss of heterozygosity; OS, overall survival.

**FIGURE 4:**
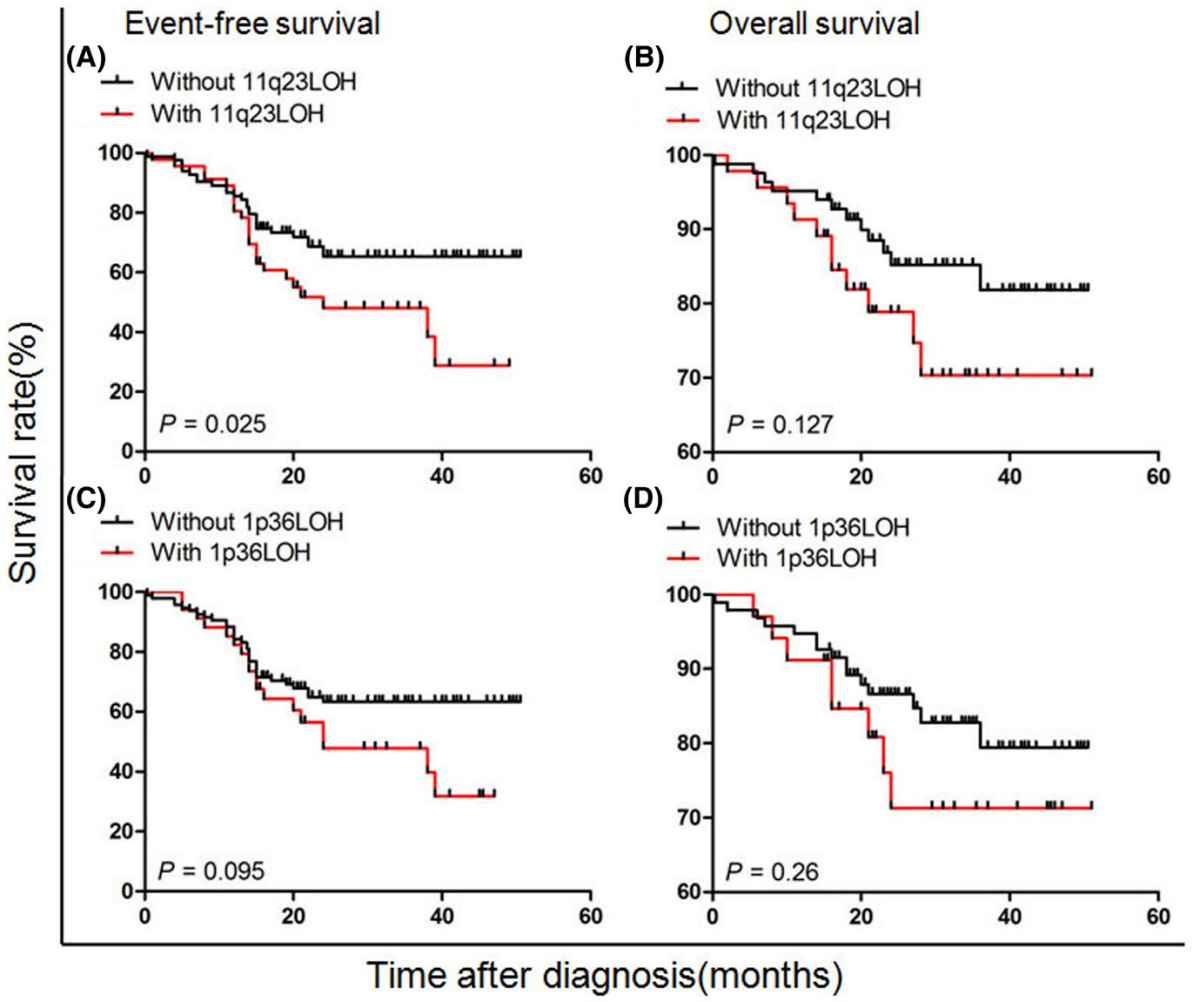
Survival rate according to 11q23 and 1p36 LOH for patients without MYCN amplification. (A, B) Event‐free survival (EFS) and overall survival (OS) stratified by 11q23 LOH. (C, D) EFS and OS stratified by 1p36 LOH. LOH, loss of heterozygosity;

However, further analysis of the subgroup of 129 patients without MYCN amplification showed that only 11q23 LOH was a powerful prognostic factor (Figure 4A,B), whereas 1p36 LOH was not (Figure 4C,D). The estimated 3‐year EFS and OS rates of patients with 11q23 LOH were 28.9 ± 1.6% and 70.3 ± 8.0, respectively, compared with 65.4 ± 5.5% (*p* = 0.025) and 81.8 ± 5.2% (*p* = 0.127) in patients who did not have 11q23 LOH (Table 4). Multivariate analysis showed that MYCN amplification was independently associated with poor EFS in all patients. 11q23 LOH was an independent prognostic factor only in those without MYCN amplification (Table 5).

**TABLE 5:** Univariate and multivariate analysis of event‐free survival of children with bone marrow metastatic neuroblastoma

Variable	Total patients (*n* = 154)				MYCN nonamplified patients (*n* = 129)			
	Univariate^a^	Multivariate^b^			Univariate^a^	Multivariate^b^		
	*p*	HR	95% CI	*p*	*p*	HR	95% CI	*p*
MYCN status (amplified vs. nonamplified)	<0.001	2.202	1.190–4.077	0.012	–	–	–	–
11q23 status (LOH vs. no loss)	0.602	NI	NI	NI	0.024	1.774	1.007–3.126	0.047
1p36 status (LOH vs. no loss)	0.002	NI	NI	NI	0.120	NI	NI	NI
LDH (≥1500 vs. <1500 IU/L)	<0.001	2.788	1.564–4.971	0.001	0.01	2.277	1.094–4.739	0.028
NSE (≥370 vs. <370 ng/ml)	0.003	NI	NI	NI	0.301	NI	NI	NI
Tumour size (≥10 vs. <10 cm)	0.113	NI	NI	NI	0.270	NI	NI	NI
Number of organs with metastasis (≥3 vs. <3)	0.174	NI	NI	NI	0.437	NI	NI	NI

Abbreviations: EFS, event‐free survival; LDH, lactate dehydrogenase; LOH, loss of heterozygosity; NSE, neuron‐specific enolase.

^a^ Univariate analysis for EFS was calculated with the use of Kaplan–Meier log‐rank test.

^b^ All factors with *p* < 0.05 in the univariate analysis were selected in the backward Cox regression model for multivariate analysis of EFS.

**FIGURE 5:**
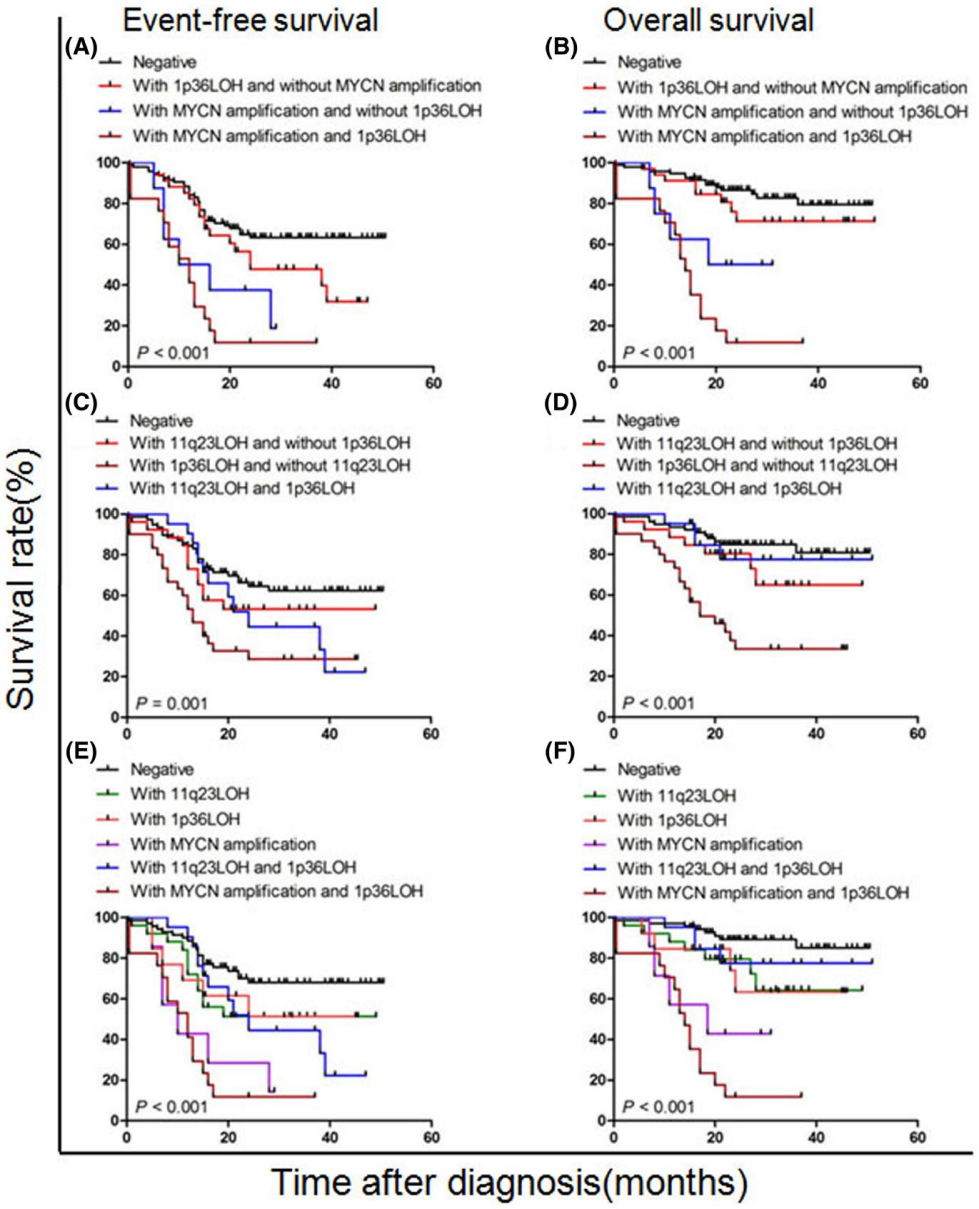
Survival rate according to combined assessment of MYCN,11q23 and 1p36 statuses. (A, B) Event‐free survival (EFS) and overall survival (OS) stratified by combined assessment according to MYCN amplification and 1p36 LOH. (C, D) Event‐free survival (EFS) and overall survival (OS) stratified by combined assessment according to 11q23 LOH and 1p36 LOH. (E, F) Event‐free survival and overall survival stratified by combined assessment according to MYCN amplification, 11q23 and 1p36 LOH. LOH, loss of heterozygosity

We further explored the prognostic value of the combination of three markers. First, patients were divided into four groups according to the status of MYCN and 1p36, as well as 1p36 and 11q23 (Figures 5A–D). There were significant differences in the EFS and OS of all groups (*p* < 0.001, *p* < 0.001, *p* = 0.001, *p* < 0.001).

Then, the 153 patients (all patients, except for one with both MYCN amplification and 11q23LOH) were further divided into six groups. Patients with MYCN amplification and 1p36LOH had the worst outcome in both EFS and OS (11.8 ± 7.8% and 11.8 ± 7.8%, respectively). The prognosis of patients with MYCN amplification alone was the second worst (14.3 ± 13.2% and 42.9 ± 18.7%, respectively). There was no difference in survival between the 1p36 LOH alone and 11q23 LOH alone groups (3‐year EFS 51.3 ± 14.6% vs. 51.3 ± 10.1%; 3‐year OS 63.5 ± 15.0% vs. 64.3 ± 11.8%). The six groups differed significantly in survival (both *p* < 0.001) (Figure 5E,F).

## 4 DISCUSSION

The roles of MYCN amplification and 1p36 and 11q23 LOH in identifying ultra high‐risk individuals remain unclear. Our study regarding the associations of the above three abnormalities with the survival of BM metastasis NB suggests that patients with both MYCN amplification and 1p36LOH had the worst survival rate and poorer clinical features, making up the ultra high‐risk group. In addition, we also found in that 11q23 LOH was an independent prognostic factor for patients without MYCN amplification, whereas 1p36LOH was not an independent prognostic factor regardless of MYCN amplification.

In recent years, multiple groups have attempted to define a subgroup of high‐risk patients with the worst outcome. The term ultra high‐risk has been used increasingly. Approximately 10%–19% of high‐risk patients are considered to be ultra high‐risk.^16^ There is almost no potential benefit for them with the current high‐risk treatment protocols. More innovative approaches need to be discovered.^17^ Therefore, identifying this subpopulation as early as possible would allow us to direct experimental therapies. In our study, 17 cases were considered to be ultra high‐risk among the 146 high‐risk patients, accounting for 11.6%. The median EFS and OS times were 12 and 14 months, respectively. However, there is no universal consensus on the definition of ultra high‐risk. Some authors believe that the survival time of ultra high‐risk patients must be shorter than 18 months or that the 5‐year EFS should be <10%–15%.^18^, ^19^

Genetic aberrations are an important aspect to guide risk stratification. However, to date, many studies have only divided patients according to MYCN gene status. In fact, the outcomes of MYCN‐amplified patients are also heterogeneous. For example, Kushner BH revealed two subgroups in MYCN‐amplified high‐risk patients, with one group having early disease progression and the other surviving for a long time,^20^ suggesting that there are other genetic aberrations attributed to the development and progression of high‐risk NB. In addition, Yuya Saito et al. showed that aggressive treatment, such as high‐dose chemotherapy and autologous HSCT, may diminish the aggressiveness of cancer with MYCN amplification.^21^ However, the results may not be applicable because other genetic factors, such as 1p and 11q deletions, which may also affect prognosis, were not taken into account.

Our study considered the status of MYCN, 11q23 and 1p36 LOH. They were present in 16.2%, 30.5% and 33.1% of patients, respectively, which was consistent with previous reports.^13^, ^22^ Here, we found that only one patient presented with both MYCN amplification and 11q23 LOH. The two markers were negatively correlated, as reported. We confirmed a positive correlation between MYCN amplification and 1p36LOH. There was no relationship between 11q23 LOH and 1p36 LOH, and no one had three markers positive at the same time.

We thought that 11q23 LOH represented a distinct genetic aberration that is different from MYCN amplification and 1p36 LOH, owing to its special clinicobiological characteristics. 11q23 LOH was more common in older patients, with a median age of 53.3 months, whereas in the MYCN amplification and 1p36 LOH groups, the median ages were 40 and 41 months, respectively. In addition, both MYCN amplification and 1p36 LOH were closely associated with poor prognostic variables, such as a high level of NSE and more events. However, 11q23 LOH was not. The unique biological characteristics give 11q23 LOH different prognostic effects. Univariate analysis showed that both MYCN amplification and 1p36LOH were closely associated with decreased EFS and OS in all patients. However, 11q23 LOH presented an unfavourable marker only in patients lacking MYCN amplification. In addition, 11q23 LOH has more of an impact on EFS than OS. Multivariate analysis confirmed that it was an independent prognostic factor for EFS. This is different from our previous result that 11q23 LOH was not an independent variable.^14^ We understood that these were two studies during different periods, and we expanded the sample size as well as the follow‐up time. In the International Neuroblastoma Risk Group (INRG) classification system, 11q deletion was associated with poor prognosis, mainly in patients with L2 or MS tumours who were fewer than 18 months old and lacked MYCN amplification.^23^ Our results suggest that in BM metastatic NB, patients with 11q23 LOH but without MYCN amplification should receive the most active treatment, regardless of age.

Although 1p36 LOH was closely associated with poor outcome in all patients, multivariate analysis showed that it was not an independent prognostic factor regardless of MYCN amplification. We thought this was mainly due to the overlap between 1p36 LOH and MYCN amplification. However, several groups have indicated the prognostic utility of 1p36 LOH in low‐risk or intermediate‐risk patients.^24^, ^25^ As there was only one intermediate‐risk patient in our study, statistical analysis could not be performed. It was reported that MYCN amplification is associated with 1p36 LOH, which is harboured in a proximal tumour suppressor region.^26^ Patients with both MYCN amplification and 1p36 LOH had the worst outcome. We thought there may be both distal and proximal suppressor regions lost with 1p36. One contributes to the initiation of NB, and the other leads to MYCN amplification.

In our study, FISH was used to detect three markers at diagnosis simultaneously.

This makes up for the deficiencies of other studies using different methods. It was reported that both PCR and Southern blotting can be used to detect specific chromosomal abnormalities.^27^ However, the sample is easily contaminated for PCR and more DNA is needed for Southern blotting. FISH is considered to be the standard method, especially for the detection of MYCN amplification. Our previous research proved that FISH has high sensitivity and accuracy in detecting genetic markers at diagnosis in BM metastatic NB, in which primary tumours are unavailable.^28^ In addition, the obvious image and commercial kits make it easier for clinical application.

## 5 Conclusions

In conclusion, our data indicate that patients with both MYCN amplification and 1p36 LOH had the worst outcome, indicating an ultra high‐risk group. 11q23 LOH was an independent prognostic factor for patients without MYCN amplification, whereas 1p36 LOH was not an independent marker, regardless of MYCN amplification. However, there are some limitations in the present study. First, the high‐risk group comprised approximately 95% of patients, making the results not universal. Second, the treatment of high‐risk patients was not uniform: <50% received transplantation, some received immunotherapy and the effect of different treatment modalities on prognosis was not taken into account. Third, how these recommendations altered treatment and whether long‐term survival changed have not been explored.

## CONFLICT OF INTEREST

The authors declare that there are no competing interests associated with the manuscript.

## AUTHORS' CONTRIBUTIONS

Zhi‑Xia Yue, Xiao‑Li Ma and Yan Su designed the study. Zhi‑Xia Yue and Xiao‑Li Ma performed the experiments, collected the data and wrote the manuscript. Tian‑Yu Xing, Chao and Shu‑Guang Liu helped to collect the samples. Wen Zhao, Qian Zhao, Xi‑Si Wang and Yan Su recruited the patients. Xiao‑Li Ma reviewed the final manuscript and take primary responsibility for the article. All authors read and approved the final manuscript.

## ETHICS APPROVAL

This research was approved by the Beijing Children's Hospital Institutional Ethics Committee (No. 2016–65).

## CONSENT FOR PUBLICATION

All authors agreed to publish.

## Data Availability

Data and material will be available upon corresponding author approval. All data analysed for this study are included in the manuscript.
